# 124. Implementation of IV Push Antibiotics for Outpatients During a National Fluid Shortage Following Hurricane Maria

**DOI:** 10.1093/ofid/ofab466.326

**Published:** 2021-12-04

**Authors:** Kruti J Yagnik, Hala Saad, Cylaina Bird, Steven Brown, Kristin Alvarez, Norman Mang, Frederick Cerise, Kavita Bhavan

**Affiliations:** 1 UT Southwestern Medical Center, Dallas, Texas; 2 University of Texas Southwestern Medical Center, Dallas, Texas; 3 UT Southwestern, Dallas, TX; 4 Parkland Hospital and Health System, Dallas, Texas; 5 PHHS, Dallas, Texas; 6 Parkland Health & Hospital System, Dallas, TX

## Abstract

**Background:**

Prior to the introduction of intravenous (IV) drip infusion, most IV drugs were delivered in a single bolus. However, with the advent of new agents, IV drip infusions became the standard for all medication delivery. In September 2017, Hurricane Maria made landfall in Puerto Rico and took a devastating toll. As Puerto Rico is the largest supplier of IV fluid bags, this lead to a worldwide fluid bag shortage. The outpatient antimicrobial therapy program (OPAT) was significantly impacted by the fluid shortage and this required effective stewardship at the Parkland Health and Hospital System in order to serve a largely uninsured and under-insured patient population.

**Methods:**

Parkland pharmacists evaluated all self-administered antimicrobials for viability of administration as an IV single bolus push (IV-push) instead of a mini-bag infusion (IV-drip infusion). These medications were transitioned to IV-push for patient care. Data was gathered on patient demographics, 30-day readmission rates, mortality, discharge teaching satisfaction, patient satisfaction, and cost evaluation.

**Results:**

113 treatment courses were self-administered using the IV-push method and were compared to 102 self-administered courses using the IV drip infusion method, over the same time course. Individuals using IV-push had a statistically significant decrease in hospital length of stay as compared to those using IV-drip infusion. The 30-day readmission rate, emergency department visits, and mortality were similar between groups. Patient satisfaction was greater with IV-push (96% preferring). The shift to IV-push via the S-OPAT program saved 504 liters of normal saline, which along with a reduction in supplies and drug costs, resulted in an additional savings of &43,652 over a 6-month period.

**Conclusion:**

The abrupt IV fluid shortage following a natural disaster challenged clinicians to think differently about standard practices. This led to implementation of a high value care model that is sustainable without affecting safety, efficacy, or efficiency. Given the cost savings, increased patient satisfaction, and equal clinical outcomes, the IV push model is not only a viable alternative initiated during a crisis; it is preferable in many standard situations.

**Disclosures:**

**All Authors**: No reported disclosures

